# Association of Atrial Fibrillation With an Increased Risk of Stroke and Functional Disability Following Hip and Pelvic Fractures

**DOI:** 10.7759/cureus.100543

**Published:** 2026-01-01

**Authors:** Ubaid Ur Rehman Khizir, Muhammad Hannan Asghar, Adeel Ur Rehman, Muhammad Subhan Sajjad, FNU Shehryaar, Sanjay Kumar, Maryam Mazhar

**Affiliations:** 1 Emergency Medicine, Evercare Hospital Lahore, Lahore, PAK; 2 Accident and Emergency Medicine, Niazi Medical & Dental College, Sargodha, PAK; 3 Neurosurgery, Punjab Institute of Neurosciences, Lahore, PAK; 4 Cardiology, Punjab Institute of Cardiology (PIC), Lahore, PAK; 5 Anatomy, Amna Inayat Medical College, Lahore, PAK; 6 Cardiology, Suleman Roshan Medical College and Hospital, Tando Adam, PAK; 7 Emergency Medicine, Niaz Ghani Hospital, Sahiwal, PAK

**Keywords:** atrial fibrillation, fracture, patient satisfaction, rehabilitation, risk factors

## Abstract

Background: Atrial fibrillation (AF) is a common arrhythmia in elderly patients and is strongly associated with increased risk of ischemic stroke, disability, and mortality.

Objective: The objective of the study is to evaluate the association of AF with ischemic stroke, functional disability, and mortality in patients presenting with hip and pelvic fractures.

Methods: This case-control study was conducted at Niazi Medical and Dental College, Sargodha, Pakistan, from January 2024 to January 2025. This study included 273 patients aged ≥50 years admitted with hip or pelvic fractures, of whom 137 had AF and 136 did not. Baseline demographics, comorbidities, and fracture characteristics were recorded. AF was confirmed via electrocardiography (ECG) or prior medical history. The primary outcome was the incidence of ischemic stroke within 90 days.

Results: The mean age of the overall cohort was 71.8 ± 8.9 years, with 59.3% being female. Compared to patients who did not have AF, those with AF were older and had higher rates of hypertension, coronary artery disease, and previous strokes. Ischemic stroke occurred in 12.4% of AF patients compared to 1.5% of non-AF patients within 90 days (p = 0.001). AF was independently associated with stroke (adjusted odds ratio (OR) = 5.2, 95% confidence interval (CI): 2.0-13.6, p < 0.001) and poor functional outcome (modified Rankin Scale (mRS) ≥ 4 at 90 days: adjusted OR = 2.1, 95% CI: 1.3-3.5, p = 0.002). At 90 days, 38.0% of AF patients were functionally independent (Barthel Index 80), compared to 56.6% of non-AF patients (p = 0.003). Mortality at 90 days was also higher in AF patients (12.4% vs. 4.4%, p = 0.021).

Conclusion: AF significantly increases the risk of ischemic stroke, functional disability, and short-term mortality in patients with hip and pelvic fractures. AF should be considered a critical prognostic factor in fracture management, and integrated multidisciplinary strategies, including optimized anticoagulation and early rehabilitation, are needed to improve outcomes.

## Introduction

Fractures are a potentially severe complication after stroke that causes poorer functional outcomes and increased morbidity. The risk of a fracture within the first year following stroke is 3%-6% [1]. Stroke survivors have a two- to fourfold higher risk of experiencing a hip fracture compared with non-stroke subjects [2]. Atrial fibrillation (AF) represents the most common sustained cardiac arrhythmia with an increasing prevalence with age. In infirmities beyond 80 years, about one in 10 people has AF, making it a priority socio-medical matter [3]. This cardiac arrhythmia is manifested by chaotic atrial movement, causing irregularities in ventricular conduction that, in turn, cause poor hemodynamics and stasis in the left atrial area. To a large extent, this pathophysiology increases the risk of thromboembolic complications, most prominently ischemic stroke [4]. It is estimated that AF raises stroke risk approximately fivefold, and strokes that occur in patients with AF tend to be worse, with higher mortality, recurrence, and permanent disability rates following stroke than those with other causes of stroke [5]. In addition to stroke, AF has been associated with cognitive decline, exercise intolerance, and systemic embolism, which together progressively lead to functional decline. In line with this cardiovascular disease burden, there is the hip and pelvic fracture problem, which is another big health crisis, particularly in the elderly [6]. The prevalence of osteoporosis, sarcopenia, and predisposition to falls in the elderly makes hip fractures the most common reason to be admitted, undergo an operation, and experience long-term impairment. The WHO estimates that hip fractures cost millions of disability-adjusted life years (DALYs) to the world, with a mortality rate as high as 20%-30% in the first year after the injury [7]. Healing following hip or pelvis fractures is quite inconsistent, but functional autonomy hardly ever returns to normal, and most patients end up in long-term care sites or institutions. Notably, fractures propounded in fragile older populations are not unique; in fact, such patients have a history of several comorbidities, such as cardiovascular disease and AF [8].

The interdependence of AF with fracture outcome is becoming a major point of interest in recent literature. Indeed, research that has compared the incidence of hip and pelvic fractures with the presence or absence of AF demonstrates that AF alone increases the risk [9]. First, anticoagulants can easily result in fall-related hemorrhage and fractures, and it is therefore necessary to decrease the risk of a stroke in AF patients. Second, one of the most common complications of AF is impaired cardiac output, decreased cerebral perfusion, and the occurrence of episodes of syncope or dizziness, which make people more likely to fall [10]. Third, chronic systemic inflammation and hormonal imbalances, as well as risk factors common to AF and skeletal fragility, including advanced age, hypertension, and diabetes, might be biological concealers between AF and skeletal fragility [11]. One meta-analysis recently reported that AF was associated with around a 1.7-fold increased risk of hip fracture regardless of a history of traditional risk factors for osteoporosis [12].

Objective

The basic aim of the study is to evaluate the association of AF with ischemic stroke, functional disability, and mortality in patients presenting with hip and pelvic fractures.

## Materials and methods

This case-control study was conducted at Niazi Medical & Dental College, Sargodha, Pakistan, from January 2024 to January 2025. The sample size was calculated using the WHO sample size calculator for comparing two proportions, with a 95% confidence interval (CI) and 80% power. Based on prior literature, the expected incidence of ischemic stroke was taken as 12% in patients with AF and 5% in those without AF, giving an absolute risk difference of 7%. To enhance feasibility while retaining sufficient power for statistical analysis, the final adjusted target sample size was set at 273 patients, with approximately 137 in the AF group and 136 in the non-AF group.

Inclusion criteria

The inclusion criteria include patients aged 50 years or older who were admitted with radiologically confirmed hip or pelvic fractures. Both male and female patients were eligible for inclusion, regardless of whether they had a prior diagnosis of AF. Additionally, only patients who provided written informed consent were included in the study.

Exclusion criteria

Patients with preexisting severe neurological disabilities unrelated to stroke, such as Parkinson's disease or advanced dementia, were also excluded due to the potential impact of these conditions on the study outcomes. Furthermore, patients with polytrauma involving multiple organ systems were excluded, as the complexity of their injuries could interfere with the evaluation of the primary outcomes.

Data collection

Age, sex, comorbidities (hypertension, diabetes, coronary artery disease, and chronic obstructive pulmonary disease (COPD)), medication use (anticoagulants, antiplatelets), and fracture type (intracapsular, extracapsular, acetabular, or pelvic ring fracture) were all gathered in addition to baseline clinical data. Twelve-lead electrocardiography (ECG) or Holter monitoring confirmed AF. Neuroimaging (CT/MRI brain) and clinical evaluation were used to support the incidence of stroke. At discharge and at the three-month follow-up, functional disability was assessed using the Barthel Index and modified Rankin Scale (mRS) [13]. The study's primary objective was to determine whether AF and the risk of ischemic stroke following a hip or pelvic fracture are linked. The Barthel Index and the mRS were used to measure functional disability following the fracture. Mortality was also assessed at the time of hospital discharge and three months after follow-up.

Statistical analysis

Data were analyzed using SPSS version 23.0 (IBM Corp., Armonk, NY, US). Continuous variables such as age and hospital stay were expressed as mean ± standard deviation, and categorical variables such as gender, comorbidities, and presence of AF were presented as frequencies and percentages. A Chi-squared test was used to assess associations between categorical variables. An independent t-test was applied for continuous data. To evaluate the association between AF and the primary outcomes of ischemic stroke, functional disability, and mortality, multivariate logistic regression was performed. This analysis adjusted for potential confounders, including age, gender, comorbidities (hypertension, diabetes, and coronary artery disease), medication use (anticoagulants, antiplatelets), and fracture type. The odds ratios (ORs) with 95% CIs were calculated to assess the strength and significance of these associations. A p-value of <0.05 was considered statistically significant.

## Results

A total of 273 patients with hip and pelvic fractures were included, of whom 137 had AF and 136 did not. The mean age of the overall cohort was 71.8 ± 8.9 years, with AF patients being significantly older than non-AF patients (74.2 ± 7.5 vs. 69.4 ± 9.1 years). Women comprised 59.3% of the study population, with a slightly higher proportion in the AF group. Comorbidities were more prevalent among AF patients, including hypertension (70.8% vs. 54.4%), coronary artery disease (36.5% vs. 19.1%), and prior stroke or transient ischemic attack (TIA) (15.3% vs. 6.6%) (Table 1).

**Table 1:** Baseline Characteristics of Patients With Hip and Pelvic Fractures (n = 273) SD: standard deviation; TIA: transient ischemic attack; COPD: chronic obstructive pulmonary disease; AF: atrial fibrillation

Variable	AF group (n = 137)	Non-AF group (n = 136)	Total (n = 273)
Age (years), mean ± SD	74.2 ± 7.5	69.4 ± 9.1	71.8 ± 8.9
Female, n (%)	85 (62.0)	77 (56.6)	162 (59.3)
Hypertension, n (%)	97 (70.8)	74 (54.4)	171 (62.6)
Diabetes mellitus, n (%)	62 (45.3)	52 (38.2)	114 (41.7)
Coronary artery disease, n (%)	50 (36.5)	26 (19.1)	76 (27.8)
Prior stroke/TIA, n (%)	21 (15.3)	9 (6.6)	30 (11.0)
COPD, n (%)	29 (21.2)	19 (14.0)	48 (17.6)

The incidence of ischemic stroke within 90 days was markedly higher in AF patients compared to those without AF (12.4% vs. 1.5%, p < 0.001). After adjustment for confounders, AF remained an independent predictor of post-fracture stroke with an OR of 5.2 (95% CI: 2.0-13.6, p < 0.001) (Table 2).

**Table 2:** Incidence of Stroke Within 90 Days CI: confidence interval; OR: odds ratio; AF: atrial fibrillation

Outcome	AF group (n = 137)	Non-AF group (n = 136)	Total (n = 273)	p-value
Ischemic stroke, n (%)	17 (12.4)	2 (1.5)	19 (6.9)	<0.001*
Unadjusted OR (95% CI)	9.2 (2.1–40.3)	1	–	<0.001*
Adjusted OR (95% CI)	5.2 (2.0–13.6)	1	–	<0.001*

Functional outcomes also differed significantly between groups. At discharge, severe disability (mRS ≥ 4) was present in 45.3% of AF patients compared to 28.7% of non-AF patients (p = 0.006). At the 90-day follow-up, only 38.0% of AF patients regained functional independence (Barthel Index ≥ 80), compared with 56.6% of non-AF patients (p = 0.003) (Table 3).

**Table 3:** Functional Outcomes at Discharge and 90 Days *p < 0.05 AF: atrial fibrillation; mRS: modified Rankin Scale

Functional status	AF group (n = 137)	Non-AF group (n = 136)	Total (n = 273)	p-value
At discharge				
mRS ≤ 3 (good outcome)	75 (54.7)	97 (71.3)	172 (63.0)	0.006*
mRS ≥ 4 (severe disability)	62 (45.3)	39 (28.7)	101 (37.0)	–
At 90 days				
Barthel Index ≥ 80 (independent)	52 (38.0)	77 (56.6)	129 (47.3)	0.003*
Barthel Index < 80 (dependent)	85 (62.0)	59 (43.4)	144 (52.7)	–

Mortality outcomes further highlighted the adverse impact of AF. In-hospital mortality was significantly higher among AF patients (7.3% vs. 2.2%, p = 0.048), and by 90 days, cumulative mortality reached 12.4% in the AF group compared to 4.4% in the non-AF group (p = 0.021) (Table 4).

**Table 4:** Mortality and Complications p-values marked with an asterisk (*) were calculated using the Chi-squared test. OR: odds ratio; CI: confidence interval; AF: atrial fibrillation Statistical significance was defined as p < 0.05.

Outcome	AF group (n = 137)	Non-AF group (n = 136)	Total (n = 273)	Unadjusted OR (95% CI)	p-value
In-hospital mortality, n (%)	10 (7.3)	3 (2.2)	13 (4.8)	3.50 (0.95–12.8)	0.048*
90-day mortality, n (%)	17 (12.4)	6 (4.4)	23 (8.4)	3.07 (1.17–8.04)	0.021*
Pulmonary infection, n (%)	19 (13.9)	11 (8.1)	30 (11.0)	1.93 (0.88–4.20)	0.15
Venous thromboembolism, n (%)	8 (5.8)	4 (2.9)	12 (4.4)	2.05 (0.62–6.74)	0.25

Multivariable logistic regression confirmed AF as a strong independent predictor of both ischemic stroke (adjusted OR 5.2, 95% CI: 2.0-13.6, p < 0.001) and poor functional outcome at 90 days (adjusted OR 2.1, 95% CI: 1.3-3.5, p = 0.002) (Table 5).

**Table 5:** Multivariable Logistic Regression for Predictors of Post-fracture Stroke and Poor Functional Outcome TIA: transient ischemic attack; OR: odds ratio; CI: confidence interval; mRS: modified Rankin Scale

Variable	Adjusted OR (95% CI) for stroke	p-value	Adjusted OR (95% CI) for poor functional outcome (mRS ≥ 4 at 90 days)	p-value
Atrial fibrillation	5.2 (2.0–13.6)	<0.001*	2.1 (1.3–3.5)	0.002*
Age ≥ 75 years	2.4 (1.1–5.2)	0.029*	1.8 (1.1–3.0)	0.018*
Hypertension	1.3 (0.6–2.8)	0.48	1.2 (0.7–2.0)	0.39
Diabetes mellitus	1.5 (0.7–3.2)	0.29	1.3 (0.8–2.3)	0.22
Prior stroke/TIA	2.9 (1.1–7.6)	0.035*	2.0 (1.0–4.1)	0.047*
Coronary artery disease	1.2 (0.5–2.8)	0.63	1.1 (0.6–2.0)	0.71
Male sex	1.0 (0.5–2.1)	0.94	0.9 (0.5–1.6)	0.72

## Discussion

This study demonstrates that AF is significantly associated with an increased risk of ischemic stroke and poorer functional outcomes following hip and pelvic fractures. Patients with AF had almost fivefold increased risks of stroke in comparison with patients without AF and were less likely to achieve independence within 90 days in terms of the Barthel Index and mRS scores. Besides, the prevalence of in-hospital and 90-day mortality was significantly worse in AF patients, indicating the clinical burden associated with the combination of a major osteoporotic fracture and AF. We have identified that there is clear evidence that AF is not only a predictor of cardiovascular disease but also a risk factor by itself of stroke, but not hip fracture outcomes. Previous research documented that AF is associated with an increase in the rate of hip fractures by 70% and nearly doubles the post-hip fracture mortality [14]. The current findings affirm that AF still portends a bad prognosis in the face of conventional pathologies, including hypertension, diabetes, and coronary artery disease. This indicates that AF is a direct actor affecting the prognosis of fractures, via such mechanisms as systemic inflammation, deficient hemodynamics, and predisposition to falls [15].

The elevated rates of stroke in AF patients after hip and pelvic fractures most likely reflect preoperative thromboembolic risk as well as difficulties. Options in anticoagulation of patients with fractures are frequently disrupted in the week before surgery, predisposing patients to thromboembolic complications. Moreover, lack of mobility, systemic inflammatory response to fracture, and hyperplatelet responsiveness can further increase the risk of strokes in the case of AF as well. This underscores the fine line clinicians should walk between prophylaxis of thromboembolic events and prevention of bleeding in patients in this cohort. Functional recovery was also significantly impaired among AF patients in this study [16]. At 90 days, only 38% of people with AF were functionally independent, compared to nearly 57% of people without AF. This result confirms previous research that hip fracture surgery is slowed down by cardiovascular comorbidities, particularly AF and heart failure. Functional disability may be exacerbated by the presence of AF, which can make frailty worse, make it harder to tolerate exercise, and make it more likely that perioperative complications like pneumonia and delirium will occur. Stroke events themselves further worsen rehabilitation outcomes, creating a vicious cycle of dependency and decline [17].

Large observational cohorts have shown that AF nearly doubles one-year mortality after hip fracture, which is consistent with the observed excess mortality in AF patients. In contrast to patients without AF, AF patients had nearly three times higher 90-day mortality in our study. While some of this mortality is attributable to stroke, the contribution of comorbidities such as pulmonary infections and coronary artery disease should not be underestimated. Importantly, multivariable regression demonstrated that even after adjustment, AF remained an independent predictor, highlighting the significance of this arrhythmia for fracture patients' prognoses [18]. These findings have significant clinical implications. First, patients with hip and pelvic fractures should be identified with AF as a high-risk marker, requiring closer monitoring during hospitalization and early rehabilitation. Second, anticoagulation management should be optimized, with careful risk-benefit assessment for perioperative interruption. Although more research is required to establish best practices in this delicate balance, the use of bridging strategies or the early resumption of direct oral anticoagulants may reduce the incidence of post-fracture stroke. Third, multidisciplinary care involving cardiology, orthopedics, geriatrics, and rehabilitation specialists is essential to improve outcomes in this vulnerable population [19].

Limitations

The prospective design, systematic follow-up, and utilization of standardized functional scales (Barthel Index, mRS) are among the study's strengths. However, several limitations must be acknowledged. First, the study was conducted at a single center with a modest sample size, which may limit generalizability. Second, even though key confounders were taken into account, residual confounding from unmeasured variables like age or nutritional status cannot be ruled out. Thirdly, information about anticoagulation therapy, such as how well it was followed and when it was resumed, was not always available, which may have affected the risk of stroke. Finally, the study's 90-day follow-up period necessitated additional investigation into longer-term outcomes.

## Conclusions

It is concluded that AF is strongly associated with an increased risk of ischemic stroke, greater functional disability, and higher short-term mortality among patients with hip and pelvic fractures. The presence of AF should therefore be considered a critical prognostic factor in fracture management. Early recognition, careful anticoagulation planning, and multidisciplinary care approaches are essential to mitigate these risks and improve post-fracture survival and functional recovery.
